# A novel prokaryotic vector for identification and selection of recombinants: Direct use of the vector for expression studies in *E. coli*

**DOI:** 10.1186/1475-2859-9-30

**Published:** 2010-05-11

**Authors:** Sampali Banerjee, Jitendra Kumar, Anjali Apte-Deshpande, Sriram Padmanabhan

**Affiliations:** 1 Lupin Limited, Biotechnology R & D, Gat #1156, Ghotawade Village, Mulshi Taluka, Pune-411042, India

## Abstract

**Background:**

The selection of bacterial recombinants that harbour a desired insert, has been a key factor in molecular cloning and a series of screening procedures need to be performed for selection of clones carrying the genes of interest. The conventional cloning techniques are reported to have problems such as screening high number of colonies, generation of false positives, setting up of control ligation mix with vector alone etc.

**Results:**

We describe the development of a novel dual cloning/expression vector, which enables to screen the recombinants directly and expression of the gene of interest. The vector contains Green fluorescence protein (GFP) as the reporter gene and is constructed in such a way that the *E. coli *cells upon transformation with this vector does not show any fluorescence, but readily fluoresce upon insertion of a foreign gene of interest. The same construct could be easily used for screening of the clones and expression studies by mere switching to specific hosts.

**Conclusions:**

This is the first vector reported that takes the property of colour or fluorescence to be achieved only upon cloning while all the other vectors available commercially show loss of colour or loss of fluorescence upon cloning. As the fluorescence of GFP depends on the solubility of the protein, the intensity of the fluorescence would also indicate the extent of solubility of the expressed target protein.

## Background

Gene cloning is a frequently used technique in molecular biology and there are several methods available for screening the recombinants like colony PCR screening, blue white screening, vector carrying toxic gene which gets inactivated upon insertion of any foreign gene, GFP fluorescence vectors wherein upon cloning, the GFP fluorescence disappears etc.

The method for screening of bacterial transformants that carry recombinant plasmid with the gene of interest, has become more rapid and simple by the use of vectors with visually detectable reporter genes. The blue white screen is one of the most common molecular techniques that allow detecting the successful ligation of gene of interest in vector [1]. The principle behind this technique is the genetic engineering of the *lac *operon in the bacterial host (*E. coli*) combined with subunit complementation (alpha complementation) achieved with the cloning vector.

Another approach includes the use of toxic genes like *ccdB *(used in pDEST vectors, Invitrogen), for positive selection of right clones, where the cytotoxic gene *ccdB *[2-4] gets inactivated if the target gene is inserted upstream of these toxic genes. Therefore the colonies that grow on selective media are only indicative of recombinants. The only disadvantage of this vector is that one need to clone the gene of interest into a separate expression vector for expression studies and that would require a second round of screening of the recombinants using conventional methods. The recently published *E. coli *toxic gene (TG) [5] also works in a similar fashion, although the mechanism of cytotoxic effect of TG has not yet been elucidated. For the above-mentioned methods, caution is required in selecting the expression host and the stop codon present in the target gene.

The green fluorescent protein (GFP) has emerged, in recent years, as a powerful reporter molecule for monitoring gene expression, protein localization and protein-protein interaction. The use of GFP from the jellyfish *Aequorea victoria *has been introduced as a tool for the study of gene expression and protein localization in various systems [6-9]. GFP has been expressed in bacteria, yeast, slime mold, plants, drosophila, zebra fish and in mammalian cells [10]. Inouye et al [11] have described a bacterial cloning vector with mutated *Aequorea Victoria *GFP protein as an indicator for screening recombinant plasmids. The pGREENscript A plasmid when expressed in *E. coli*, produced colonies showing yellow colour in day light and strong green fluorescence under long-UV. Inserted foreign genes are selected on the basis of loss of the fluorescence caused by inactivation of the GFP production. While GFP solubility appears to be one of the limiting factors in whole cell fluorescence, Davis and Vierstra [12] have reported about soluble derivatives of GFP for use in *Arabidopsis thaliana*.

All the above-described plasmids could also result in false positive clones, which is a major concern for researchers. In case of blue-white selection using beta galactosidase, sometimes the blue colour is lost due to chemical instability making it difficult to differentiate a recombinant versus a non-recombinant clone. Also, loss of GFP fluorescence due to medium composition is also known to lead to false positive results. To avoid these problems, we have designed a unique strategy to screen recombinants carrying the gene of interest in such a way that all the clones carrying the foreign gene would show fluorescence, with no exceptions.

In this report we describe a novel cloning/expression vector for one-step screening and expression of foreign genes. The strategy uses the cloning of gene for GFP into any expression vector with a stop codon upstream of the ORF of GFP, other than the amber stop codon. Upon induction, the GFP would not express and hence would not fluoresce due to presence of the stop codon. In frame cloning of gene of interest upstream of GFP, would then excise the initial stop codon and the resultant fusion protein would fluoresce. The gene of interest contains an amber stop codon and the recombinant screening is carried out in an amber suppressor *E. coli *strain. For expression studies, the same clone can be used for checking expression in a non amber suppressor *E. coli *host. This is the first report of a vector where *in situ *screening of the transformants for the presence of recombinants is possible without any false positive results.

## Results

### Modification of pBAD24M vector and cloning of GFP

**Figure 1:**
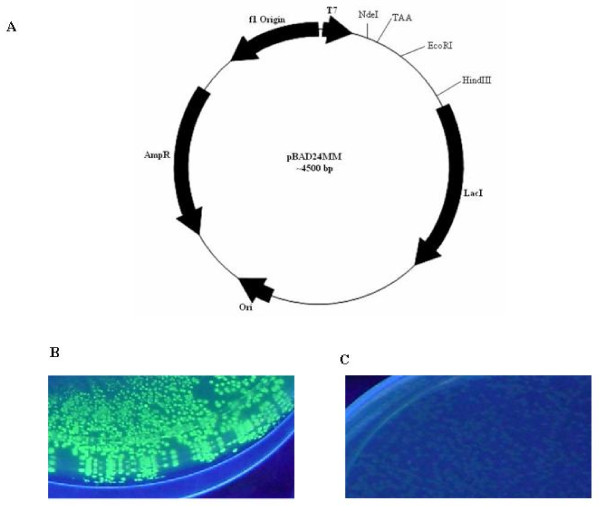
**Effect of introduction of stop codon upstream of GFP gene on the fluorescence**. A. Clone map of the pBAD24MM where a stop codon was inserted between. NdeI and EcoRI site. B and C are the fluorescence of GFP upon induction expressed from pBAD24M-GFP and pBAD24MMGFP, respectively. Note the complete loss of GFP fluorescence with pBAD24MM-GFP.

To construct the single step screening and expression vector, a stop codon (like TAA) other than the amber codon (TAG) was introduced between NdeI and EcoRI sites by site directed mutagenesis (SDM) to pBAD24M vector and the resultant construct was designated as pBAD24MM (Fig. 1A). The TAA stop codon of pBAD24MM vector does not allow expression of any foreign gene when cloned downstream of the same stop codon.

As a reporter gene, the gene for GFP was cloned into vectors pBAD24M and pBAD24MM as EcoRI/HindIII fragment. Both the constructs were transformed into BL21 cells and plated on inducer containing media (13 mM arabinose). Figure 1B shows GFP fluorescence upon UV exposure in pBAD24M while the fluorescence was completely abolished in pBAD24MM (Fig. 1C) vector due to presence of the stop codon. This result indicates that the stop codon TAA interfered with the translation of the protein as expected.

**Figure 2:**
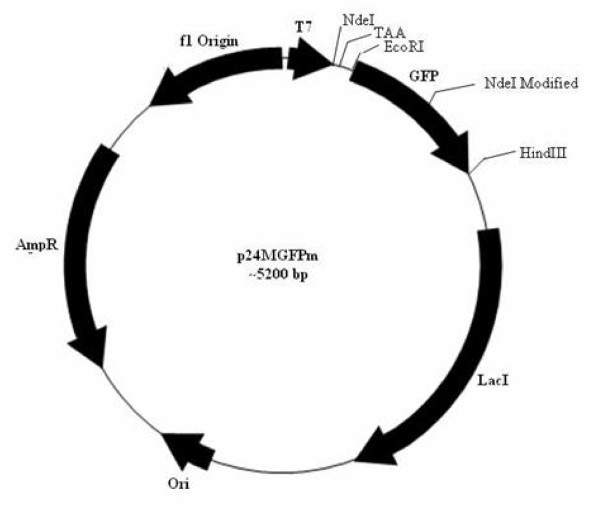
**Plasmid map of NdeI modified GFP (p24MGFPm)**. NdeI site in GFP was deleted by SDM keeping the amino acid sequence of GFP unaltered.

To clone any foreign gene at the NdeI site of the pBAD24MM-GFP vector, the internal NdeI site present in the gene for GFP ought to be removed without altering the amino acid sequence that eventually would also not affect the GFP fluorescence. The internal NdeI site of the GFP gene in the pBAD24M-GFP construct was hence mutated by SDM (data not shown) and the induction studies of such a construct showed no effect on GFP fluorescence. Such a modified GFP gene was later sub cloned into pBAD24MM vector and the resultant vector is designated as p24MGFPm (Fig. 2).

### Cloning of foreign genes in p24MGFPm vector and *in situ *screening of the recombinants

**Figure 3:**
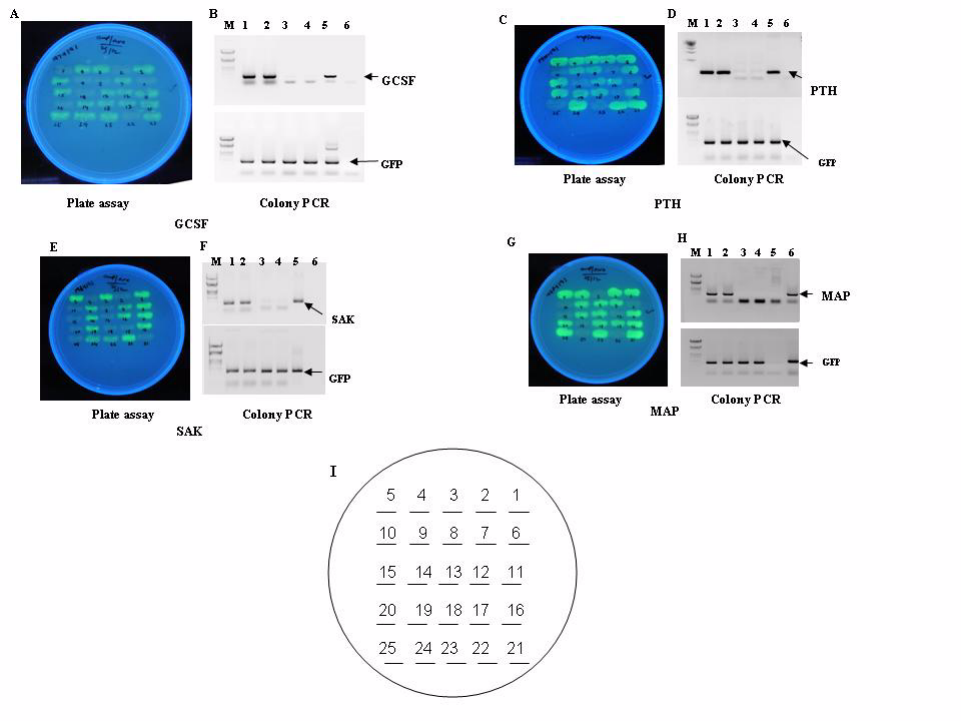
**Replica plating of randomly chosen colonies from transformed amber suppressor strain LE392 with the ligation mixtures of p24MGFPm with GCSF, PTH, SAK, MAP respectively (Please refer to methods section for details). **The transformants were plated on inducer (13 mM L+Arabinose) containing plates that indicate both glowing and non glowing colonies upon UV exposure. Colony PCR of randomly chosen two glowing and two non-glowing colonies from each plate were carried out for GFP and for genes of interest. Panel A, C, E and G show the GFP fluorescence from recombinants of p24MGFPm-GCSF, p24MGFPm-PTH, p24MGFPm-SAK, p24MGFPm-MAP from LB amp plates containing inducer (13 mM arabinose). Panel B: GCSF-GFP fusion: Lanes 1 & 2 are the PCR products from glowing colonies nos. 1 & 3 respectively and lanes 3 & 4 are the same from two non-glowing colonies nos. 2 & 5. Lanes 5 & 6 are positive and negative controls, respectively. Panel D: PTH-GFP fusion: Lanes 1 & 2 is the PCR products from glowing colonies nos. 1 & 2 respectively and lanes 3 & 4 are the same from two non-glowing colonies nos. 7 & 10. Lanes 5 & 6 is positive and negative controls, respectively. Panel F: SAK-GFP fusion: Lanes 1 & 2 are the PCR products from glowing colonies nos. 1 & 3 respectively, and lanes 3 & 4 are the same from two non-glowing colonies nos. 2 & 4. Lanes 5 & 6 are positive and negative controls, respectively. Panel H: MAP-GFP fusion: Lanes 1 & 2 are the PCR products from glowing colonies nos. 1 & 2 respectively, and lanes 3 & 4 are the same from two non-glowing colonies nos. 3 & 6. Lanes 5 & 6 are negative and positive controls, respectively. In all the cases M stands for molecular weight marker (Lambda DNA EcoRI/HindIII digest); Upper panels are gene specific PCR and Lower panels indicate GFP specific PCR. Arrows indicate corresponding PCR product. Panel I: Representative diagram showing the lay out of the clones in the replica plate.

Our objective was to use the constructed vector p24MGFPm as a cloning vehicle for screening recombinants by a unique strategy of appearance of GFP fluorescence only in recombinants carrying the target gene. All the target genes with amber stop codon (TAG) were used for the cloning and amber suppressor *E. coli *strain LE392 was used for expression of fusion proteins. Due to insertion of the target genes at NdeI/EcoRI site in frame with GFP, the stop codon TAA is removed and only the recombinants with C-terminus GFP fusion would fluoresce in an amber suppressor strain upon induction and exposure to UV light. To substantiate our concept, we carried out cloning of genes like Staphylokinase (SAK), a soluble protein from *Staphylococcus aureus *of ~15 kDa, human granulocyte colony stimulating factor (hGCSF) of 18 kDa, human parathyroid hormone (hPTH) of 4 kDa and *E. coli *methionine amino peptidase (MAP) of ~26 kDa as NdeI/EcoRI fragments in p24MGFPm vector so that the resultant clones would be C terminal GFP fusions. The recombinants upon transfer to an amber suppressor *E. coli *would glow after induction and could be selected by mere visualization under UV light. Hence, the ligation mixtures of p24MGFPm and foreign gene(s) were transformed to an amber suppressor strain LE392 and plated on LB-amp media. Following day, the colonies were replica plated on 13 mM arabinose containing media and after incubation at 30°C, plates were exposed under UV (long uv) showing some glowing and some non-glowing colonies (Fig. 3A, C, E and 3G). To confirm our hypothesis, both fluorescing and non-fluorescing colonies from all the four ligations were subjected to PCR amplification with GFP specific and gene specific primers individually. As shown in Figure 3, all glowing cells were PCR positive for GCSF (Fig. 3B, lanes 1 and 2), PTH (Fig. 3D, lanes 1 and 2), SAK (Fig. 3F, lanes 1 and 2) and MAP (Fig. 3H, lanes 1 and 2) genes and all non glowing colonies were negative for all the above gene specific PCR (Fig. 3B, D, F, H, lanes 3 and 4). On the other hand all glowing and non-glowing colonies were positive for GFP PCR (Fig. 3B, D, F, H, lower panels) as expected.

### Expression of GFP fusions in an amber suppressing strain LE392 and quantitation of GFP fluorescence

To show the expression of the fusion proteins of GFP, the plasmid DNA from all the glowing colonies of the four ligation mix namely p24MGFPm-GCSF, p24MGFPm-PTH, p24MGFPm-SAK and p24MGFPm-MAP were introduced into competent cells of LE392 and induced with 13 mM arabinose for 4 hours at 37°C.

**Figure 4:**
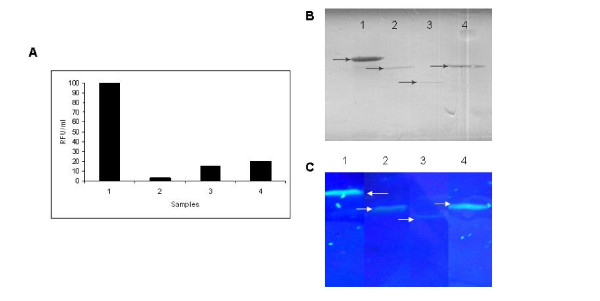
**Effect of solubility of target protein on GFP fluorescence**. A. Relative GFP fluorescence assay for GFP fusion proteins. B. Western blot of soluble fraction of GFP fusion proteins using anti-GFP antibody. Lane 1: MAP-GFP, Lane 2: GCSF-GFP, Lane 3: PTH-GFP, Lane 4: SAK-GFP. C. SDS-PAGE profile of the soluble fractions of the expressed fusion proteins followed by visualization under UV light. The induced cells expressing the fusion proteins were normalized to equal cell density and lysed by sonication. The soluble fractions were analyzed on SDS-PAGE followed by visualization upon UV exposure. Lane 1: MAP-GFP fusion (~55 kDa), lane 2: GCSF-GFP fusion protein (~41 kDa), lane 3: PTH-GFP fusion (~30 kDa), and lane 4: SAK-GFP fusion (~41 kDa). Arrows indicate the fusion proteins. Note the difference in fluorescence intensities of different fusion proteins directly reflecting the solubility of the target gene.

To gain insight into the levels of GFP expressed from all these fusions, GFP assay was carried out from all the soluble fractions of GFP fusion proteins from normalized cell density using the kit from Cell Bio labs, USA as per manufacturer's instructions along with denaturing SDS-PAGE followed by western blot. It is interesting to see that units of GFP fluorescence varied based on the solubility of the target protein (Fig. 4A). The expression of all the GFP fusion proteins in the soluble fractions was confirmed by western blot using anti-GFP antibody (Fig. 4B). The SDS-PAGE profile of the soluble fractions of the fusion proteins without sample boiling (Fig. 4C) showed that better solubility of the GFP fusions leads to higher fluorescence.

### Expression of the genes of interest in a non amber suppressing *E. coli *strain BL21

**Figure 5:**
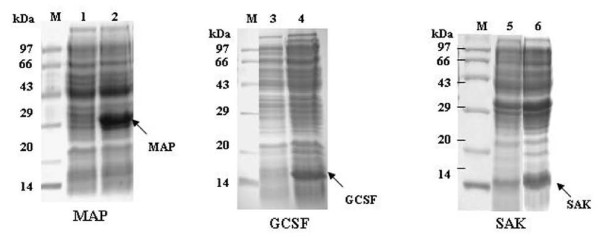
**SDS-PAGE showing protein expression from one glowing and one non-glowing clones of p24MGFPm-MAP, p24MGFPm-GCSF and p24MGFPm-SAK in non-amber suppressor strain BL21 *E. coli *cells**. M: Protein molecular marker, lanes 1, 3, 5: Expression of foreign genes from non-glowing colony containing MAP-GFP, GCSF-GFP and SAK-GFP constructs respectively. Lanes 2, 4, 6: SDS-PAGE profile of glowing colonies from each construct.

The glowing clones of GCSF, SAK and MAP along with non-glowing clones from all the experimental sets were introduced in BL21 cells (a non amber suppressor *E. coli *B strain) and expressions were induced with 13 mM L(+) arabinose. Expression of the heterologous proteins was analyzed on regular SDS-PAGE followed by Coomassie blue staining. As expected, the protein synthesis from glowing colonies was terminated at amber stop codon in all the constructs and proteins of expected molecular size for example, GCSF (18 kDa), SAK (15 kDa) and MAP (26 kDa) were seen on SDS-PAGE (Fig. 5, lanes 2). The non-glowing clones from all the sets did not express any recombinant protein since they were non-recombinants with TAA at the N terminus of the GFP gene (Fig. 5, lanes 1). The expression of PTH (~4 kDa) was not tested in this study since expression of untagged PTH in *E. coli *yields very low amounts of PTH due to its RNA and the protein instability.

## Discussion

In this study, we report the development of a dual-function prokaryotic vector to be used for *in situ *screening of recombinants as well as for expression studies. In a conventional method, the transformants are screened either by carrying out the colony PCR or plasmid preparation to confirm the recombinants. Recently, use of many toxic genes like *ccdB*, *TG *etc. have been reported for screening the recombinants. We have constructed a novel vector with GFP as a reporter gene for screening and expression of recombinants. For the first time we have successfully shown that upon insertion of any target gene upstream of GFP results in appearance of GFP fluorescence which provides direct evidence of presence of the target gene in the vector.

Earlier reports have suggested that GFP expression is affected by OmpT proteases as there are two putative OmpT protease sites in the coding region of gene for GFP [13] and with the observations that since OmpT expression is low at 28-30°C, the GFP expression is pronounced at these temperatures [14,15]. Inclusion of inhibitors of OmpT like zinc chloride and copper chloride at 0.1 to 0.5 mM final concentration also are known to reduce OmpT expression in such cells [16]. Based on these literature reports,, we carried out all our GFP fluorescence plate assays at 30°C instead of regular 37°C though we did not observe any enhancement of fluorescence intensity upon addition of metal ions. To substantiate the use of amber stop codon in the target gene for recombinant screening, we have used *E. coli *LE 392 strain which is known to be a strong amber suppressor strain.

All other commercially avaliable vectors show loss of color or loss of fluorescence that may not be unfailing while the major advantage of the vector described in this report, takes the property of color or fluorescence obtained after cloning. This unique vector would also be applicable with any other reporter genes like beta galactosidase gene, luciferase gene, DsRed protein instead of the described gene for GFP in the same vector constructed similarly. It also provides researchers to skip to set up the control ligation mix (without insert) and the dephosphorylation step (CIP or SAP step) since the religated vector would never glow and all the fluorescing colonies are indicative of only the recombinants and also indicative of correct reading frame of the inserted target gene. Since GFP fluorescence is brightest when it is expressed in soluble form [17], the intensity of the fluorescence after cloning the foreign gene would also indicate the extent of solubility of the fusion protein. We have cloned four genes namely hGCSF, hPTH, SAK and MAP as examples where GCSF is a known insoluble protein while SAK and MAP are known soluble proteins when expressed in *E. coli*. Since untagged PTH is known to be unstable in *E. coli *[18], we did not study its expression in a non-amber suppressing strain. Our plate assay data showing the least intensity with the GCSF-GFP fusion and maximum intensity with MAP-GFP along with similar data from GFP quantitation assay indicate a new possibility of use of this vector for predicting the solubility status of the foreign gene. Moreover, upon switching to a non-suppressor strain like BL21, this construct would yield proteins with no extra amino acid at the N-terminus. A prokaryotic vector indicating solubility of any foreign gene product based on cloning is the first report of its kind.

## Conclusions

In conclusion, we report a novel dual purpose vector for *in situ *recombinant screening and expression of genes in bacterial system. The fluorescence intensity of the reporter gene with the fusion partner is indicative of the solubility index for the target protein.

## Methods

### Construction of vectors and clones

#### Modification of pBAD24M vector

The pBAD24M vector [18] was modified by introducing a STOP codon other than amber codon after NdeI site using site directed mutagenesis (SDM) kit following manufacturer's (Stratagene, USA) protocol. The primers used was as follows; forward: 5' T AGC ATG ACT GGT GGA CAG **TAA **ATG GGT CGC GGA TCC GAA TTC GA 3'

Reverse: 5' TC GAA TTC GGA TCC GCG ACC CAT **TTA **CTG TCC ACC AGT CAT GCT A 3'.

The incorporated STOP is shown in bold and this modified vector was designated as pBAD24MM.

#### Cloning of GFP in pBAD24M and pBAD24MM vectors

The GFP gene was PCR amplified from pGFPuv (Clonetech) by using the following primers; forward: 5' TCC CCC ATG GTA GAA TTC AGT AAA GGA GAA GAA CTT TTC ACT 3'; reverse: 5' CCG CCG GTC GAC AAG CTT TTA TTT GTA GAG CTC ATC CAT GCC 3' and cloned into pBAD24M and pBAD24MM as EcoRI/HindIII fragment.

#### Modification of internal NdeI restriction site of the gene for GFP

Cloning of genes of interest is usually done at NdeI site of the vector so as to obtain an efficient translation initiation. As the gene for GFP contains an internal NdeI site (at 230 position), this site was removed by SDM using the following primers, forward: 5' GC TTT TCC CGT TAT CCG GAT CAC ATG AAA CGG CAT GAC 3'; reverse: 5' GTC ATG CCG TTT CAT GTG ATC CGG ATA ACG GGA AAA GC 3' without altering the amino acid (changes are shown by underline). This modification did not affect the GFP fluorescence as observed by the plate assay and the vector was designated as p24MGFPm.

#### Cloning of foreign genes like GCSF, PTH, SAK and MAP into p24MGFPm vector

All the genes were PCR amplified using synthetic genes (procured from GenScript, USA and BioServe Technologies, India) as template with Taq DNA polymerase and the PCR products were cloned into p24MGFPm vector as NdeI/EcoRI fragments. Details of GCSF and SAK PCR are given elsewhere [19,20]. For cloning of MAP gene, the PCR reaction was carried out in a two-step manner at 94°C for 5 min followed by 5 cycles of 94°C for 30 s, 50°C for 30 s and 72°C for 30 s; 25 cycles of 94°C for 30 s, 60°C for 30 s and 72°C for 30 s and final primer extension at 72°C for 5 min. The primers were: forward, 5' CCG CCGGAATTCCATATGGCTATCTCAATCAAGACCCCAGAA 3' and reverse, 5'CCGCCGGAATTCAAGCTTCTATTCGTCGTGCGAGATTATCGC 3'. For cloning the hPTH gene, the primers were, forward, 5' CCG CCG GGA TCC CAT ATG TCT GTGCTC GAG ATT CGG TTA 3' and reverse 5' CCG CCG GAA TTC AAG CTT CTA AAA ATT GTG CAC ATC CTG 3' and the PCR was carried out in two steps with annealing temperature of 43°C for 5 cycles and annealing temperature of 60°C for rest of the 25 cycles.

### GFP plate assay

All the relevant constructs were introduced into competent LE392 *E. coli *(*hsd*R514(r_k_^-^, m_k_^+^), *gln*V(*sup*E44), *try*T (*sup*F58), *lac*Y1 or Δ(*lac*IZY)6, *gal*K2, *gal*T22, *met*B1, *trp*R55) cells and plated on LB agar containing ampicillin (100 μg/ml) and 13 mM arabinose as an inducer and incubated at 30°C for 16 hrs. Subsequently the plates were exposed to UV to observe the fluorescence.

### GFP fluorescence on SDS-PAGE

GFP fluorescence for the fermentation samples was done as per the protocol described by Baird et al. [21] with little modification. Briefly, to prevent denaturation, protein samples were mixed with SDS-PAGE sample buffer and after incubating at 45°C for 10 minutes, centrifuged briefly and loaded onto SDS-PAGE. The gel was later visualised under UV light and photographs documented.

### GFP quantitation assay

GFP quantitation fluorometric kit (Cell Bio labs, USA) that measures GFP fluorescence in a fluorometer was used for relative quantitation of GFP produced from all constructs under test. The relative quantity of GFP in sample was determined by comparing its fluorescence with different samples. The kit has a detection sensitivity limit of (0.1 ng of GFP/μl). A proprietary GFP quench solution is also included for determining auto fluorescence of all the cell samples.

### Expression studies

For all expression studies, suitable *E. coli *cells were taken and grown in Luria broth (LB) at 30°C or 37°C and induced at an OD_600 _0.8-1.0 with 13 mM arabinose. After 4 hrs of additional growth at 30°C or 37°C, all the samples were analyzed on SDS-PAGE.

### Western blot

Protein samples were separated on 12% SDS-PAGE gels and electro blotted onto nitro cellulose membrane. The membrane was blocked with 3% BSA in TBST (10 mM Tris.Cl with 150 mM NaCl and 0.1% Tween 20) for 1 h at 37°C. The membrane was then incubated for 1 h at room temperature with rabbit anti-GFP antibody (diluted in TBST with 0.3% BSA). After three washes with TBST buffer, the membrane was incubated for 1 h with alkaline phosphatase conjugated anti rabbit IgG (diluted in TBST with 0.3% BSA). The membrane was washed three times and specific protein was visualized by adding BCIP/NBT solution (Bangalore Genei, India).

## Competing interests

The authors declare that they have no competing interests.

## Authors' contributions

SB generated the idea of using amber suppressor strain for in-situ screening of recombinants and participated in the design and coordination of the experiments along with drafting the manuscript. JK carried out cloning of most of the constructs described. AD constructed the vector pBAD24MM. SP conceived the concept, participated in the study design and critically evaluated the data generated. All the authors read and approved the final manuscript.
